# Improved Water-Pump

**Published:** 1847-08

**Authors:** 


### IMPROVED WATER-PUMP.

As having a direct and important bearing upon *hygiène*, we would call the attention of our readers to an improvement made by an enterprising and ingenious citizen of Louisville in the water-pump. This pump combines the *forcing* with the *suction* principle, and is capable of elevating water from a cistern or even a deep well to the top of an ordinary two-story building. It is thus invaluable not only in case of fire, against which it may be used as a fire-engine, but also for the purpose of supplying water for bathing in any apartment of the house. This pump has been fully tested in Louisville, and is superseding all others. So indispensable is an abundant supply of cold water to health and comfort, that we feel secure of the thanks of our readers for this notice, which will lead many of them to avail themselves of a truly valuable invention. The manufacturer is Mr. Chapman Warner.

